# Intraperitoneal paclitaxel plus intravenous fluorouracil, leucovorin, oxaliplatin (FOLFOX) and nivolumab for gastric cancer with peritoneal metastasis: results from the IPLUS Phase II study

**DOI:** 10.1007/s10120-026-01741-y

**Published:** 2026-04-19

**Authors:** So Hyun Kang, Jin Won Kim, Eunju Lee, Mira Yoo, Dongjae Jeon, Woochan Park, Minsu Kang, Hyeon Jeong Oh, Sa-Hong Min, Young Suk Park, Tae-Han Kim, Yoon Jin Lee, Ji-Won Kim, Sang-Hoon Ahn, Yun-Suhk Suh, Keun-Wook Lee, Hye Seung Lee, Hyung-Ho Kim

**Affiliations:** 1 Department of Surgery, Seoul National University Bundang Hospital, Seoul National University College of Medicine, Seongnam, Korea; 2 Department of Internal Medicine, Seoul National University Bundang Hospital, Seoul National University College of Medicine, Seongnam, Korea; 3 Department of Surgery, Chung-Ang University Gwangmyeong Hospital, 110 Deokan-ro, Gwangmyeong-si, Gyeonggi-do Korea; 4 Department of Pathology, Seoul National University Bundang Hospital, Seoul National University College of Medicine, Seongnam, Korea; 5 Department of Surgery, Asan Medical Center, Seoul, Korea; 6 Department of Radiology, Gangnam Severance Hospital, Seoul, Korea; 7 Department of Surgery, Samsung Medical Center, Seoul, Korea; 8 Department of Pathology, Seoul National University Hospital, Seoul National University College of Medicine, Seoul, Korea

**Keywords:** Stomach neoplasm, Peritoneal neoplasms, Chemotherapy

## Abstract

**Background:**

Gastric cancer with peritoneal metastasis (PM) carries a poor prognosis, with median overall survival (mOS) of 9–11 months using standard systemic chemotherapy. Intraperitoneal (IP) chemotherapy has shown promise, but its effect still remains unclear. This study aims to show the efficacy and safety of IP paclitaxel with systemic FOLFOX ± nivolumab in gastric cancer patients with PM.

**Methods:**

This single-arm, open-label Phase II IPLUS trial evaluated biweekly IP paclitaxel (60 mg/m^2^) plus systemic FOLFOX ± nivolumab in 22 patients with histologically confirmed gastric cancer and PM, aged 20–80 years, and no other hematogenous metastasis. Co-primary endpoints were one-year OS and progression-free survival (PFS) rate, with secondary endpoints including mOS, median PFS (mPFS), response rates, conversion surgery rate, and safety per CTCAE v5.0.

**Results:**

From January 2021 to August 2022, 22 patients received a mean of 16.0 ± 13.6 cycles. One-year OS and PFS rates were 86.4% and 63.6%, respectively, with mOS of 20.2 months (95% CI 17.2–32.9) and mPFS of 13.1 months (95% CI 12.2–25.7). Conversion surgery was achieved in 40.9%, with mOS of 32.9 months for the conversion surgery group. Of patients with measurable lesion, RECIST v1.1 responses (n = 5) showed an objective response rate of 40.0% and disease control rate of 80.0%.

**Conclusion:**

IP paclitaxel plus FOLFOX ± nivolumab demonstrates encouraging survival outcomes and tolerability in gastric cancer with PM with high conversion surgery rates.

**Supplementary Information:**

The online version contains supplementary material available at 10.1007/s10120-026-01741-y.

## Introduction

Gastric cancer (GC) remains a global health challenge, ranking fifth in both incidence and cancer-related mortality worldwide [1]. Peritoneal metastasis (PM) is the most frequent pattern of dissemination in advanced GC, occurring in 10–40% of patients and indicating a dismal prognosis [2, 3]. Standard systemic chemotherapy, typically fluoropyrimidine- and platinum-based regimens, yields median overall survival (mOS) rates of 9–11 months for gastric cancer with peritoneal metastasis (GCPM), underscoring the limited efficacy of intravenous approaches in penetrating the peritoneal cavity [4, 5]. The most recent major clinical trials using immunotherapy for metastatic GC such as the Checkmate 649 and ATTRACTION-4 trials both showed no superior effect of nivolumab in increasing the survival of patients with PM [6, 7]. The blood-peritoneum barrier restricts drug diffusion to peritoneal lesions, necessitating innovative strategies to improve therapeutic outcomes [8].

Intraperitoneal (IP) chemotherapy has emerged as a promising modality to address this challenge by delivering high concentrations of chemotherapeutic agents directly into the peritoneal cavity, achieving prolonged exposure to cancer cells while minimizing systemic toxicity [4]. Preclinical evidence demonstrates a nearly 2500-fold increase in peritoneal tissue exposure with IP administration compared to intravenous delivery, supporting its theoretical advantage [9]. A recent Asian expert consensus statement, developed using GRADE-based evidence appraisal and a Delphi voting process among 30 experts across four Asian countries, provides practical recommendations for normothermic intraperitoneal and systemic (NIPS) therapy in gastric cancer with peritoneal metastasis—covering diagnosis and patient selection, NIPS regimens, intraperitoneal port management, and peri-/post-conversion surgery strategies [10]. The PHOENIX-GC trial, a notable Phase III study, compared IP paclitaxel plus systemic S-1 to systemic S-1 plus cisplatin, reporting mOS of 17.7 versus 15.2 months, respectively, though statistical significance was not achieved (P = 0.080) [11]. Although several clinical studies have reported encouraging results, with a recent meta-analysis indicating a pooled mOS of 14.5 months for IP chemotherapy in GC with PM [12], the role of IP chemotherapy for GCPM patients remains under investigation.

The IPLUS Phase I trial, conducted at Seoul National University Bundang Hospital, evaluated the safety and feasibility of IP paclitaxel combined with systemic FOLFOX (fluorouracil, leucovorin, and oxaliplatin) with the addition of nivolumab in GC patients with PM [13]. This dose-escalation study (40–80 mg/m^2^ IP paclitaxel) established a recommended phase II dose of 60 mg/m^2^, demonstrating a tolerable safety profile with manageable toxicities (e.g., neutropenia, nausea) and a mOS of 16.6 months (95% CI 16.6-N/A) among 13 patients. Notably, 23.1% of patients underwent conversion surgery, with preliminary evidence suggesting improved survival in this subgroup, aligning with findings that IP chemotherapy may serve as induction therapy for potentially resectable disease. Building on these findings, the phase II of the IPLUS trial was designed to assess the efficacy and safety of biweekly IP paclitaxel at 60 mg/m^2^ combined with systemic FOLFOX ± nivolumab in GCPM patients.

## Methods

### Study design

The Phase II IPLUS trial was a single-arm, open-label study conducted at Seoul National University Bundang Hospital to evaluate the efficacy and safety of intraperitoneal (IP) paclitaxel combined with systemic FOLFOX (fluorouracil, leucovorin, and oxaliplatin) ± nivolumab in patients with GCPM. Building on the phase I dose-escalation study, which established 60 mg/m^2^ as the recommended phase II dose of IP paclitaxel [13], this trial aimed to assess the one-year overall survival (OS) and one-year progression-free survival (PFS) as the co-primary endpoint in a larger cohort. The study was approved and annually reviewed by the institutional review board (B-1802–448-006) and the Ministry of Food and Drug Safety (MFDS) of Korea (201,800,197), and conducted in accordance with the Declaration of Helsinki and Good Clinical Practice guidelines. All patients signed a written informed consent after given thorough explanation of the study.

### Patient eligibility

Eligible patients were adults aged 20–80 years with histologically confirmed adenocarcinoma of gastric origin (primary or recurrent) and radiologically or laparoscopically confirmed PM. Inclusion criteria included: no prior systemic chemotherapy, Eastern Cooperative Oncology Group (ECOG) performance status of 0–2, HER-2 negative status, and adequate organ function as defined by absolute neutrophil count ≥ 1,500/mm^3^, hemoglobin ≥ 8.0 g/dL, platelet count ≥ 100,000/mm^3^, aspartate aminotransferase (AST) and alanine aminotransferase (ALT) ≤ 100 U/L, total bilirubin ≤ 2.0 mg/dL, and creatinine clearance ≥ 50 mL/min. Patients were required to be evaluated and deemed suitable for FOLFOX ± nivolumab by a multidisciplinary outpatient clinic.

Exclusion criteria included: presence of other major medical diseases or concurrent malignancies, presence of other distant organ metastasis (except Krukenberg tumors or para-aortic lymph node involvement), contraindications to 5-fluorouracil (5-FU), oxaliplatin, leucovorin, or paclitaxel, pregnancy or breastfeeding, or plans for childbirth during the study period. Compared to phase I, eligibility criteria were maintained to ensure consistency, with no additional restrictions on peritoneal carcinomatosis index (PCI) scores.

### Intervention: chemotherapy

Patients received biweekly cycles of IP paclitaxel at 60 mg/m^2^ combined with systemic FOLFOX ± nivolumab [13]. On Day 1 of each cycle, IP paclitaxel was administered in 1L of normal saline (adjusted based on ascites volume) over 30–60 min via an indwelling peritoneal access port (Celsite, B-Braun, Germany). Concurrently, systemic FOLFOX ± nivolumab was delivered as follows: oxaliplatin 100 mg/m^2^ intravenously (IV) over 2 h, leucovorin 100 mg/m^2^ IV over 2 h, and 5-FU 2400 mg/m^2^ IV over 46 h via continuous infusion. Nivolumab use was determined by shared decision-making between the physician and the patient regardless of PD-L1 expression as an additional non-reimbursed first-line therapy. Treatment was repeated every 14 days until disease progression, unacceptable toxicity, or patient withdrawal. Dose reductions (e.g., 25% reduction in IP paclitaxel or FOLFOX components) were permitted by the physician’s discretion per Common Terminology Criteria for Adverse Events (CTCAE) v5.0 guidelines.

### Assessments

Pre-treatment evaluations included complete blood counts, liver and renal function tests, tumor markers (CEA, CA 19-9, CA 125), HER-2 status, and imaging (computed tomography [CT] with optional magnetic resonance imaging [MRI] or positron emission tomography [PET]). Baseline ECOG status and PCI score (via diagnostic laparoscopy or imaging) were recorded. During treatment, patients underwent biweekly clinical assessments, including toxicity monitoring and laboratory tests. Tumor response was evaluated by CT/MRI every 6–8 weeks (approximately every 3 cycles).

### Intervention: surgical

Second-look diagnostic laparoscopy was offered to patients with clinical response or stable disease after 6–9 cycles to assess PCI score changes and obtain peritoneal biopsies for peritoneal regression grading score (PRGS) analysis. Conversion surgery was performed when there was major or complete response of peritoneal dissemination according to PRGS in second look diagnostic laparoscopy, and grossly R0 resection including the primary tumor was possible. For conversion surgery, D2 lymph node dissection (LND) was performed. Either total or distal gastrectomy was done, making sure all surgical margins were negative. Minimally invasive approach was allowed at surgeon’s preference. All adjacent organs with possible tumor invasion was also excised. Post-treatment follow-up occurred every 2–3 weeks for survival and adverse event (AE) monitoring until death or study closure.

### Outcomes

The co-primary endpoints were one-year OS rate and one-year PFS rate. OS was measured from treatment start to death from any cause, and PFS was defined as the time from treatment initiation to disease progression per Response Evaluation Criteria in Solid Tumors (RECIST) v1.1 or death from any cause. Secondary endpoints included mOS, mPFS, objective response rate (ORR) defined as the proportion of patients achieving complete response (CR) or partial response (PR), disease control rate (DCR) encompassing CR, PR, and stable disease (SD), conversion surgery rate, and AEs per CTCAE v5.0. PRGS in the peritoneal biopsy samples of patients undergoing second-look laparoscopy was also assessed and scored from 1 (complete regression) to 4 (no response) [14].

### Statistical analysis

The sample size for the phase II IPLUS trial was determined with assistance from the Medical Research Center of Seoul National University Bundang Hospital (SNUBH) to ensure adequate power to detect a clinically meaningful improvement in one-year OS. The calculation assumed a baseline one-year OS of 45% for systemic FOLFOX ± nivolumab chemotherapy in gastric cancer with peritoneal metastasis, derived as a historical control from studies which evaluated FOLFOX ± nivolumab in advanced gastric cancer [15]. The experimental arm, based on Chan et al. [16], targeted a one-year OS of 72.2%, as observed in their phase II study of IP paclitaxel plus systemic XELOX (capecitabine and oxaliplatin) for GCPM. Using a two-sample proportion test with a significance level (α) of 0.05, a Type II error (β) of 0.20 (yielding 80% power), the required sample size was calculated as 21 patients. Accounting for an anticipated dropout rate of 20%, the total sample size was adjusted to 25 patients.

One-year OS and PFS rates were estimated using the Kaplan–Meier method, and 95% CIs were calculated using Greenwood’s formula with a log–log transformation. Median OS and PFS were also analyzed. ORR was calculated among patients with measurable target lesions, whereas DCR was calculated in the full cohort and included patients with non-target lesions only categorized as non-CR/non-PD. The conversion surgery rate was analyzed descriptively. Subgroup analyses compared outcomes between patients undergoing resection versus those who did not, using the log-rank test for survival endpoints. AEs were tabulated by grade and cycle. Statistical analyses were performed using R (version 3.4.1, http://www.r-project.org/) and the survival package (http://CRAN.R-project.org/package=Survival) was used, and *p* < 0.05 was considered statistically significant.

## Results

### Patient characteristics

From January 2021 to August 2022, 25 patients were enrolled in the phase II IPLUS trial at Seoul National University Bundang Hospital. Three patients dropped out before completing the study due to free will, leaving 22 evaluable patients. Baseline characteristics are presented in Table 1. The median age was 50.5 years (range 28–70 years), with 36.4% (n = 8) male and 63.6% (n = 14) female. The majority of patients had an ECOG performance status of 1 (86.4%, 19/22), with 3 (13.6%) having ECOG grade 2. All patients had histologically confirmed gastric adenocarcinoma with PM confirmed by baseline diagnostic laparoscopy, with a mean baseline PCI score of 19.6 ± 13.7 (range 3–39). Histological analysis according to WHO classification revealed poorly differentiated adenocarcinoma in 4 (18.2%), poorly differentiated adenocarcinoma with signet ring cells in 11 (50.0%), poorly cohesive carcinoma in 5 (22.7%), moderately differentiated adenocarcinoma in moderately differentiated adenocarcinoma with signet ring cells in 1 (4.5%) patient. Microsatellite stability status showed that 90.9% (20/22) were microsatellite stable (MSS), while 1 patient (4.5%) had microsatellite instability-low (MSI-L) and 1 patient (4.5%) had microsatellite instability-high (MSI-H). Nivolumab was administered to 13 (59.1%) patients,. Most patients (95.5%, 21/22) had primary disease, with 1 (4.5%) having recurrent disease after previous gastric cancer treatment.

**Table 1:** Patient demographics and baseline tumor characteristics

	N = 22
Age (years)	50.5 ± 12.5
Sex	
Male	8 (36.4%)
Female	14 (63.6%)
Baseline peritoneal carcinomatosis index score	19.6 ± 13.7
Primary or recurrent	
Primary	21 (95.5%)
Recurrent	1 (4.5%)
ECOG^a^ performance status	
Grade 1	19 (86.4%)
Grade 2	3 (13.6%)
WHO histologic classification	
Poorly differentiated	4 (18.2%)
Poorly differentiated with SRC^a^	11 (50.0%)
Poorly cohesive carcinoma	5 (22.7%)
Moderately differentiated with SRC	1 (4.5%)
Moderately differentiated	1 (4.5%)
Microsatellite stability status	
MSS (microsatellite stable)	20 (90.9%)
MSI-L (microsatellite instability-low)	1 (4.5%)
MSI-H (microsatellite instability-high)	1 (4.5%)
Nivolumab use	13 (59.1%)

^a^abbreviations: ECOG = Eastern Cooperative Oncology Group, SRC = signet ring cells

### Survival analysis and tumor response

**Fig. 1:**
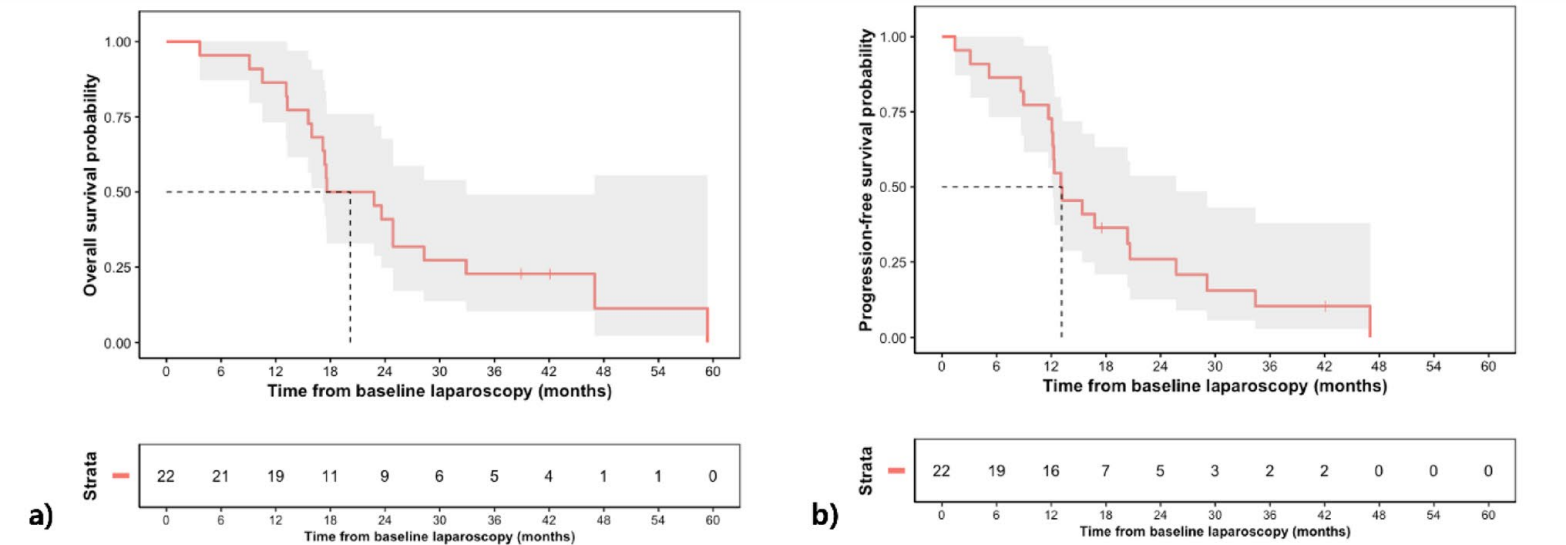
Kaplan–Meier graphs of gastric cancer patients with peritoneal metastasis after intraperitoneal paclitaxel and systemic FOLFOX ± nivolumab. **a** overall survival, **b** progression-free survival

**Fig. 2:**
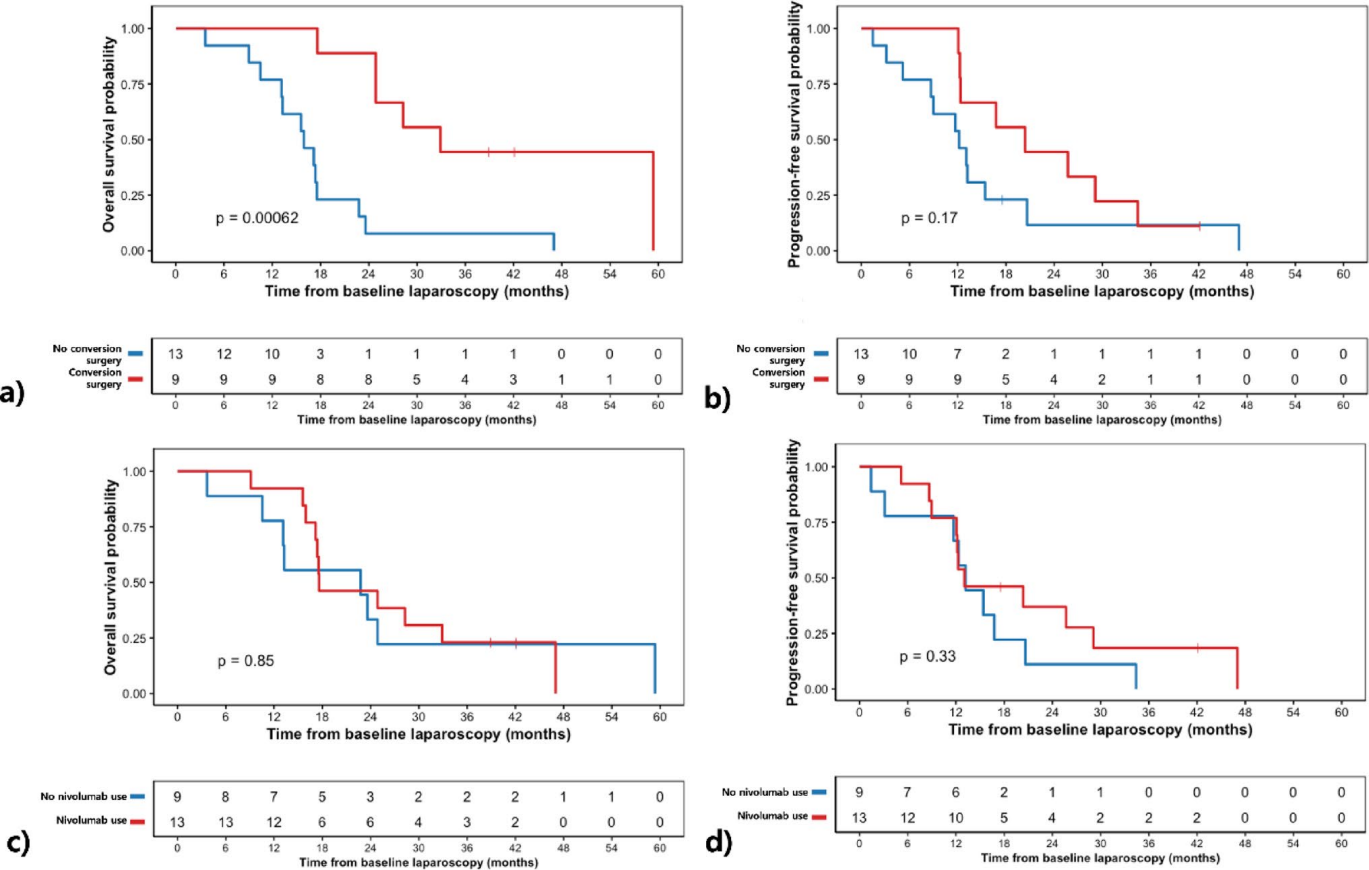
Kaplan–Meier graphs according to subgroups. **a** overall survival comparing conversion surgery group and no conversion surgery group, **b** progression-free survival comparing conversion surgery group and no conversion surgery group, **c** overall survival comparing nivolumab group and FOLFOX only group, **d** progression-free survival comparing nivolumab group and FOLFOX only group

Patients received a mean of 16.0 ± 13.6 cycles with a median of 10 cycles (range 3- 63). The primary endpoints one-year OS and PFS rates were 86.4% (19/22; 95% CI 63.4–95.4) and 63.6% (14/22; 95% CI 40.3–79.9) respectively (Fig. 1). The mOS was 20.2 months (95% CI 17.2–32.9), and mPFS was 13.1 months (95% CI 12.2–25.7). Subgroup analysis was made by grouping the patients according to conversion surgery (Fig. 2a, b) and nivolumab treatment (Fig. 2c, d). For patients who underwent conversion surgery (n = 9), mOS was 32.9 months (95% CI 24.9–N/A) and mPFS was 20.4 months (95% CI 12.3—N/A), compared to 15.9 months (95% CI 13.1-N/A) and 12.2 months (95% CI 8.7—N/A) for the no conversion surgery group (n = 13; P =  < 0.001 for OS, P = 0.17 for PFS). In the exploratory subgroup analysis of the use of nivolumab as an additional optional therapy, OS and PFS did not differ significantly according to nivolumab exposure (P = 0.855 for OS and P = 0.332 for PFS; Fig. 2c, d).

Among the 22 patients, 5 (22.7%) had measurable target lesions and were evaluable for target-lesion response by RECIST v1.1: PR in 2 (40.0%), SD in 2 (40.0%), and PD in 1 (20.0%), yielding an ORR of 40.0% (2/5). The remaining 17 patients (77.3%) had non-target lesions only and were categorized as non-CR/non-PD (rather than non-evaluable) in the absence of CR or unequivocal PD, per RECIST v1.1. When non-CR/non-PD was included, the DCR in the full cohort was 95.5% (21/22). Conversion surgery was performed in 40.9% (9/22) of patients, including 4 total/laparoscopic total gastrectomies (TLTG/LTG), 3 laparoscopic distal gastrectomies (LDG), 1 open distal gastrectomy (DG), and 1 open TG with diaphragm resection (Table 2). Cytology conversion from positive to negative occurred in 83.3% (5/6) of patients with baseline positive cytology.

**Table 2:** Treatment response after chemotherapy

	N = 22
Average number of cycles	16.0 ± 13.6
Median cycles (range)	10 (3–63)
Tumor response per RECIST	
Patients with measurable target lesions	5 (22.7%)
Partial response^a^	2 (40.0%)
Stable disease^a^	2 (40.0%)
Progressive disease^a^	1 (20.0%)
ORR (PR)^b^	2 (40.0%)
Patients without measurable target lesions	17 (77.3%)
Non-CR/non-PD^c^	17 (100.0%)
DCR (PR + SD)^d^	21 (95.5%)
Conversion surgery rate	9 (40.9%)
Cytology conversion (n = 6)^e^	5 (83.3%)
Pathologic CR	2 (40.0%)
One-year OS	86.4% (95% CI 63.4–95.4)
One-year PFS	63.6% (95% CI 40.3–79.9)
Median OS (months)	20.2 (95% CI 17.2–32.9)
Median PFS (months)	13.1 (95% CI 12.2–25.7)

^a^Calculated within patients with measurable lesions (n = 5). ^b^ORR was calculated among patients with measurable target lesions (n = 5). ^c^Calculated within patients without measurable lesions. ^d^DCR was calculated in the full cohort (N = 22) and includes non-CR/non-PD in the numerator. ^e^mong patients with positive baseline cytology

### Adverse events and safety profiles

The biweekly regimen of IP paclitaxel (60 mg/m^2^) plus FOLFOX was generally well-tolerated, with no treatment-related deaths reported among the 22 patients. Table 3 shows the AEs assessed per the CTCAE v5.0. Among hematologic toxicities, neutropenia was the most frequent severe event, with grade 3 occurring in 45.5% (10/22) and grade 4 in 31.8% (7/22) of patients. Leukopenia was also common, with grade 3 in 13.6% (3/22), while anemia and thrombocytopenia were predominantly mild to moderate (grade 1–2), with grade 3 incidences of 9.1% (2/22) and 4.5% (1/22), respectively. There was no case of febrile neutropenia during this study. Non-hematologic toxicities included significant nausea, with grade 3 in 40.9% (9/22), and anorexia, with grade 3 in 13.6% (3/22), indicating gastrointestinal effects as a notable burden. Peripheral neuropathy, likely oxaliplatin-related, affected 45.5% (10/22) at grade 2 and 4.5% (1/22) at grade 3, while oxaliplatin hypersensitivity (grade 3) occurred in 13.6% (3/22). Liver enzyme elevations (e.g., ALT grade 3 in 27.3%, 6/22; AST grade 3 in 13.6%, 3/22) were frequent but manageable. Progression-related complications, such as bowel obstruction and gastric outlet obstruction, necessitated surgical interventions in some cases, but no severe IP specific complications were noted. IP chemoport related complications included 1 case with mild swelling, 1 case of skin decolorization, 2 cases of cellulitis that required oral antibiotics, and 1 case that needed outpatient-based wound closure.

**Table 3:** Toxicity profiles of patients who received intraperitoneal Paclitaxel with systemic FOLFOX ± nivolumab

Category	Toxicity	Grade 1	Grade 2	Grade 3	Grade 4
Hematologic	ALP elevation	12	1	1	0
	ALT elevation	12	0	6	0
	AST elevation	16	1	3	0
	Anemia	10	7	2	0
	Hyperbilirubinemia	2	2	0	0
	Leukopenia	8	8	3	0
	Neutropenia	1	2	10	7
	Thrombocytopenia	7	3	1	0
Non-hematologic	Anorexia	5	12	3	0
	Constipation	1	1	0	0
	Diarrhea	0	2	0	0
	Fatigue	7	4	0	0
	Nausea	4	7	9	0
	Oxaliplatin hypersensitivity	0	0	3	0
	Peripheral neuropathy	6	10	1	0
	Vomiting	7	3	3	0
	Wound	2	3	0	0

### Second-look diagnostic laparoscopic and conversion surgery

Second-look diagnostic laparoscopy was performed in 16 patients (72.7%) after a median of 9 cycles (range 3–22) with findings detailed in Supplementary Table 1 (Online Resource). Baseline PCI scores averaged 19.6 ± 13.7 (range 3–39), decreasing to a mean of 9.5 ± 12.9 post-treatment (range 0–39). Notably, 9 patients (56.3%) achieved a post-treatment PCI of 0, indicating complete macroscopic regression of peritoneal disease. These patients subsequently underwent conversion surgery with 3 laparoscopic distal gastrectomy, 3 laparoscopic total gastrectomy, 1 open distal gastrectomy, 1 open total gastrectomy with partial diaphragm resection, and 1 open total gastrectomy with adrenalectomy. All the conversion surgery procedures were performed with D2 lymph node dissection, and all have achieved grossly R0 including surgical margins. All adjacent organs that were suspicious of invasion were also excised. One patient received palliative laparoscopic total gastrectomy due to symptoms, and this was not considered as a conversion surgery. Cytology conversion from positive to negative was observed in 85.7% (6/7) of patients with baseline positive cytology. PRGS was assessed across four quadrants (RUQ, RLQ, LUQ, LLQ), ranged from 1 (complete regression) to 4 (no response). PRGS 1 was predominant, observed in 9 patients (56.3%) across all quadrants where evaluable (see Supplementary Table 1 in Online Resource).

### Risk factor evaluation

The Cox proportional hazards model was used to assess predictors of OS in the 22 patients, with results presented in Supplementary Table 2 (Online Resource) as an exploratory analysis of survival outcomes. All variables included in the analysis, including age (HR 0.39; 95% CI 0.08–1.78; P = 0.67), male sex (HR 0.98; 95% CI 0.93–1.03; P = 0.81), baseline PCI score (HR 1.02; 95% CI 0.98–1.07; P = 0.33), and nivolumab use (HR 0.67; 95% CI 0.21–2.09; P = 0.33), were not statistically significant predictors of OS.

## Discussion

The results of the phase II part of the IPLUS trial demonstrate that biweekly IP paclitaxel at 60 mg/m^2^ combined with systemic FOLFOX ± nivolumab is a safe and efficacious regimen for GCPM patients, yielding promising survival outcomes and a high conversion surgery rate. In a cohort of 22 evaluable patients, the study achieved a mOS of 20.2 months (95% CI 17.2–32.9) and a mPFS of 13.1 months (95% CI 12.2–25.7), substantially exceeding historical systemic chemotherapy benchmarks of 9–13 months for MST [5–7]. These results build on the phase I IPLUS findings, which reported a mOS of 16.6 months and mPFS of 9.6 months [13], suggesting enhanced efficacy with an extended treatment duration (mean 16.0 ± 13.6 cycles) and the recommended IP paclitaxel dose at 60 mg/m^2^.

When comparing the IPLUS trial results to other major IP chemotherapy trials, several key differences emerge. The PHOENIX-GC trial, a randomized phase III study, compared IP paclitaxel plus S-1 and intravenous paclitaxel versus cisplatin plus S-1 in patients with gastric cancer and peritoneal metastasis, reporting median survival times of 17.7 versus 15.2 months, respectively (P = 0.08). The DRAGON-01 trial, another significant phase III study, evaluated neoadjuvant intraperitoneal and intravenous paclitaxel plus S-1, demonstrating improved overall survival with a median survival time of 19.4 months (95% CI 17.1–22.9). The PIPS-GC trial investigated IP paclitaxel combined with systemic SOX (S-1 plus oxaliplatin), reporting one-year progression-free survival of 28.6% (95% CI 8.86–92.2%) and one-year overall survival of 51.4% (95% CI 23.6–100%). In contrast to these studies, the IPLUS trial presents unique features that distinguish it from previous IP paclitaxel investigations. First, this is the first study to evaluate the feasibility of adding nivolumab into an IP paclitaxel regimen for gastric cancer with peritoneal metastasis. Second, the use of FOLFOX as the systemic chemotherapy component differs from the predominant S-1-based regimens employed in PHOENIX, DRAGON-01, and PIPS-GC trials. Although the IPLUS study showed better survival outcomes, including a median survival time of 20.2 months, one-year overall survival of 86.4%, and conversion surgery rate of 40.9%, it is crucial to acknowledge the inherent limitations of cross-trial interpretations due to differences in study design, patient populations, systemic chemotherapy backbones, and criteria for conversion surgery.

The efficacy of this regimen is further highlighted by the benefits and potential synergistic effects of the systemic FOLFOX ± nivolumab and IP paclitaxel regimen. FOLFOX is a well-established first-line treatment for GC, demonstrating response rates of 38–50% and mOS of 9–13 months in metastatic settings [5]. Patients with GCPM often have ascites, decreased bowel motility, and abdominal pain which all lead to poor oral intake. Unlike SOX or XELOX, FOLFOX is a totally intravenous regimen that can be used for patients with poor oral intake. Several studies have noted the efficacy of FOLFOX as first line therapy for patients with GCPM [17, 18]. The addition of paclitaxel through the IP method reduces the toxicity while possibly giving a maximal cytotoxic effect on the target cancer cells. IP paclitaxel, with its pharmacokinetic advantage of achieving high peritoneal concentrations and prolonged retention [19], complements FOLFOX by targeting peritoneal lesions directly, potentially enhancing cytotoxicity through synergistic mechanisms. In a human cell line analysis by Tanaka et al. [20], the cell lines that were exposed to paclitaxel before oxaliplatin showed maximal synergistic effect which is the same as the treatment schedule in the IPLUS Trial. Also, nivolumab is now approved in most GC treatment guidelines as first-line therapy in combination with other 5-FU and platinum based first-line regimens [21, 22]. The inclusion of nivolumab in 59.1% of the cohort may have further potentiated the results of the study, though its impact was not significant in Cox analysis.

A major result of this study is the 40.9% conversion surgery rate (9/22 patients), which was significantly higher than the 23.1% in phase I and surpassing the pooled rate of 16% reported in a recent meta-analysis of IP chemotherapy for GC with PM [12]. This finding aligns with prior evidence that resection following IP chemotherapy can substantially enhance survival, as demonstrated in Phase II trials like Saitoh et al. [23], where IP paclitaxel and systemic SOX facilitated conversion surgery in 45% of patients, yielding a mOS of 17.7 months. Moreover, in the AIO-FLOT3 trial of limited metastatic gastric/gastroesophageal junction cancer [24], patients who proceeded to surgical resection after neoadjuvant FLOT chemotherapy achieved a median overall survival of 31.3 months versus 15.9 months in those who did not undergo surgery. A similar pattern is seen in the international CONVO-GC-1 study [25], which analyzed over 1,200 patients undergoing conversion therapy across Japan, Korea, and China. In that cohort, patients who achieved R0 resection following systemic chemotherapy had a median survival time of 56.6 months, substantially exceeding the outcomes of R1 or R2 resections. Collectively, these studies suggest that, in carefully selected patients who respond to induction therapy, achieving R0 resection is associated with prolonged survival; however, differences in study populations, metastatic burden, and surgical selection criteria preclude causal inference that surgery itself is a major determinant of long-term survival.

The safety profile of the regimen was acceptable and manageable, with no treatment-related deaths and low rates of severe toxicities. Notably, the IP administration of paclitaxel minimized systemic absorption, which significantly reduced concerns about toxicity typically associated with triplet chemotherapy regimens. Despite combining IP paclitaxel with systemic FOLFOX ± nivolumab, the limited systemic absorption of intraperitoneally administered paclitaxel meant that the anticipated additive toxicities from this intensive combination were not realized. The manageable toxicities observed, including grade 3–4 neutropenia (45.5–31.8%), nausea (40.9%), and peripheral neuropathy (4.5%), were consistent with the IPLUS phase I findings [13] and other trials of IP chemotherapy [11, 26, 27]. This favorable safety profile demonstrates that the pharmacokinetic advantage of IP delivery—achieving high local concentrations while maintaining low systemic exposure—allows for the safe administration of this intensive multi-drug regimen. Progression-related complications required surgical intervention in some cases, but no severe IP port-specific issues were noted in this study, aligning with the low complication rates reported in recent reviews.

Several limitations temper the findings of this study. The single-arm design precludes direct comparison to systemic treatments or other IP regimens, limiting causal inference about superiority. The small sample size (N = 22) may underpower detection of rare AEs, subgroup effects, and baseline PCI variability (3–39). The limited RECIST v1.1 evaluability (5/22 patients) restricts tumor response assessment, with most patients non-evaluable due to PM’s diffuse nature. This notes for the development of better diagnostic tools for noninvasive evaluation of PM. Because nivolumab was optional and administered in a non-randomized manner based on shared decision-making during a non-reimbursed period, its incremental contribution cannot be determined from this study. Exploratory analyses by nivolumab exposure should therefore be interpreted descriptively. Therefore, the present data support the feasibility of the overall regimen rather than demonstrating a causal incremental benefit of nivolumab. Also, macroscopic assessment of PCI scores in the post-treatment setting remains imperfect. First, post-treatment PCI was assessed using the same stepwise laparoscopic mapping as at baseline. Because residual peritoneal nodularity or thickening after chemotherapy may reflect fibrosis/scarring rather than viable tumor, any suspicious seeding-like lesion was biopsied by PCI region; regions were assigned a score of 0 when final histopathology showed no malignant cells. Nevertheless, sampling error may have led to under- or overestimation of residual disease.

Despite these limitations, the IPLUS phase II trial holds significant impact and importance in the management of GCPM. By demonstrating a median overall survival of 20.2 months and a 40.9% conversion surgery rate with biweekly IP paclitaxel plus systemic FOLFOX ± nivolumab, this study surpasses historical benchmarks and recent IP chemotherapy trials [6, 7, 11, 17, 18, 26, 27]. The high conversion rate, facilitated by the regimen’s ability to reduce peritoneal disease burden, underscores its role as a bridge to achieving curative surgery, a critical unmet need in PM patients. Furthermore, the use of FOLFOX, contrasting with predominant S-1-based regimens, offers a tolerable, effective systemic option, minimizing gastrointestinal toxicity in PM, and suggests a synergistic mechanism with IP paclitaxel that warrants further exploration. Lastly, this is currently the only study that reports the use of bidirectional chemotherapy using immunotherapeutic agents for gastric cancer. These results not only inform clinical practice by providing a feasible treatment strategy but also sets the stage for future Phase III trials to validate its findings, potentially reshaping guidelines for PM management and improving patient outcomes globally.

## Conclusion

The phase II of the IPLUS trial highlights the potential of IP paclitaxel plus FOLFOX ± nivolumab, demonstrating encouraging survival outcomes and high conversion surgery rates in GCPM patients, with tolerable toxicity.

## Supplementary Information

Below is the link to the electronic supplementary material.

Supplementary Material 1
