# Supplementary material for: Intraperitoneal paclitaxel plus intravenous fluorouracil, leucovorin, oxaliplatin (FOLFOX) and nivolumab for gastric cancer with peritoneal metastasis: results from the IPLUS Phase II study
Source: Gastric Cancer. 2026 Apr 19;29(4):788–97. doi: 10.1007/s10120-026-01741-y (PMC13315462; doi:10.1007/s10120-026-01741-y)
Supplement: Supplementary file 1 — Supplementary Material 1 [file 10120_2026_1741_MOESM1_ESM.docx]

**Supplementary Tables**

**Supplementary Table 1. Details of the second-look diagnostic laparoscopy**

| **Case No.** | **Sex** | **Age** | **Baseline PCI** | **Second Look PCI** | **Surgery** | **Treatment cycles before second look laparoscopy** | **Cytology conversion** | **PRGS Grade (RUQ)** | **PRGS Grade (RLQ)** | **PRGS Grade (LUQ)** | **PRGS Grade (LLQ)** |
| --- | --- | --- | --- | --- | --- | --- | --- | --- | --- | --- | --- |
| Case 02 | F | 55 | 23 | 0 | laparoscopic distal gastrectomy | 9 | NA | 1 | 1 | 1 | 1 |
| Case 07 | F | 43 | 10 | 0 | laparoscopic distal gastrectomy | 9 | NA | 1 | 1 | 1 | 1 |
| Case 10 | F | 70 | 8 | 0 | laparoscopic total gastrectomy | 12 | Yes | 1 | NA | 1 | NA |
| Case 13 | M | 52 | 4 | 0 | open distal gastrectomy | 8 | Yes | 1 | 1 | 1 | 1 |
| Case 17 | F | 47 | 14 | 23 | palliative total gastrectomy | 9 | Yes | 3 | 4 | 4 | 2 |
| Case 18 | M | 37 | 39 | 0 | laparoscopic total gastrectomy | 10 | NA | 1 | 1 | 1 | 1 |
| Case 19 | M | 62 | 7 | 0 | laparoscopic distal gastrectomy | 7 | NA | 1 | 1 | 1 | 1 |
| Case 20 | F | 42 | 3 | 1 | open total gastrectomy with diaphragm partial resection | 7 | NA | 2 | 1 | 1 | 1 |
| Case 23 | M | 70 | 7 | 0 | open total gastrectomy adrenalectomy | 8 | Yes | 1 | 1 | 1 | 1 |
| Case 24 | F | 46 | 39 | 0 | laparoscopic total gastrectomy | 10 | Yes | 2 | 2 | 2 | 2 |

**Supplementary Table 2. Cox Proportional Hazards Model of risk factors for overall survival**

| Variables | Hazard Ratio | Lower 95% CI | Upper 95% CI | p-value |
| --- | --- | --- | --- | --- |
| Age | 0.386 | 0.084 | 1.775 | 0.669 |
| Male Gender | 0.981 | 0.933 | 1.032 | 0.812 |
| Baseline PCI | 1.022 | 0.977 | 1.068 | 0.860 |
| Nivolumab use | 0.668 | 0.214 | 2.087 | 0.325 |
